# Creative and expressive practices as coping strategies among working women during the COVID-19 pandemic: a qualitative study

**DOI:** 10.3389/fpsyg.2026.1750496

**Published:** 2026-05-08

**Authors:** Kasturi Bai Munusamy, Mohd Nazri Bin Abdul Rahman, Amira Najiha Binti Yahya, Hamed Khazaei

**Affiliations:** 1 Pusat Kecemerlangan Kesihatan Mental Kebangsaan, Kementerian Kesihatan Malaysia, Kuala Lumpur, Malaysia; 2 Scipeline, Toronto, ON, Canada

**Keywords:** coping strategies, creative expression, expressive arts practices, pandemic stress, qualitative research, women's mental health

## Abstract

**Introduction:**

The COVID-19 pandemic intensified stress among working women in Malaysia as they managed overlapping professional, domestic, emotional, and financial pressures. This study explored how working women experienced pandemic-related stress and how they used expressive arts practices as part of their coping efforts.

**Methods:**

Guided by a qualitative interpretivist approach, the study involved semi-structured, in-depth interviews with 31 working women in Malaysia aged 21 to 60 who remained employed during the pandemic and engaged in expressive activities such as drawing, painting, dance, clay work, and writing. Data were analyzed using reflexive thematic analysis.

**Results:**

The findings showed that participants experienced stress through interconnected challenges, including mental and emotional turbulence, difficulties with work-life integration, financial insecurity, and job uncertainty. Participants described expressive arts practices as helpful for non-verbal emotional expression, self-exploration, physical and mental relief, positive emotions, and psychological stabilization.

**Discussion:**

Expressive arts practices were generally self-initiated and adapted to participants' personal routines and emotional needs rather than delivered as formal therapist-led interventions. The study suggests that creative and expressive practices may serve as accessible coping resources for working women during crisis periods and highlights the importance of recognizing gendered stress experiences in workplace and mental health support initiatives.

## Introduction

The COVID-19 pandemic created profound disruptions to daily life and intensified psychological distress across populations. Stress, anxiety, uncertainty, and emotional exhaustion became common features of the pandemic experience, reflecting the broader mental health burden associated with prolonged health threats, social isolation, and economic instability (Rehman et al., 2020). Stress occurs when individuals appraise circumstances as taxing or exceeding their resources and threatening their wellbeing (Bienertova-Vasku et al., 2020). Its consequences are far-reaching, affecting both physical and mental health. Chronic stress, in particular, has been associated with a wide range of adverse outcomes, including cardiovascular problems, mental health disorders, burnout, absenteeism, and reduced job satisfaction (McEwen and Karatsoreos, 2020; Boamah et al., 2022; Hofmann et al., 2025). In work settings, excessive workload and sustained performance pressures can intensify stress and undermine work-life balance, especially when individuals are required to manage responsibilities within constrained and uncertain conditions (Delisle, 2020; Vandenabeele et al., 2025; Madigan and Kim, 2021). Within this broader context, working women were particularly vulnerable to pandemic-related stress. Women play a vital role in sustaining families and communities, and their ability to live free from chronic stress is essential to their personal and social wellbeing (Naganathan et al., 2021). During the pandemic, however, many working women had to navigate the dual burden of paid employment and domestic responsibilities under highly unstable conditions. In Malaysia, this burden was intensified by cultural and social expectations that often position women as primary carers, thereby making the management of work and family roles more stressful (Duong et al., 2020; Nappo, 2020). National reports also indicated a substantial rise in mental health concerns among women, while lockdown conditions contributed to increased domestic pressures and abuse, further exacerbating psychological strain (Women's Aid Organisation, 2024; Buselli et al., 2020). Previous studies have documented the psychological and physiological effects of stress in the aftermath of the pandemic, yet the specific stressors experienced by working women, particularly those linked to the overlap of professional and domestic responsibilities, remain insufficiently explored (Hilton, 2021; Buselli et al., 2020; Ali et al., 2022). As these pressures intensified, coping and emotional regulation became increasingly important. Individuals often relied on coping strategies that helped them manage uncertainty, express emotion, and preserve psychological balance during a period of prolonged disruption. In this context, creative and expressive activities gained relevance as accessible and personally meaningful forms of coping. The non-verbal nature of arts-based activities can offer individuals a way to process stress and anxiety when these experiences are difficult to articulate verbally. Existing literature suggests that art-related practices may support emotional expression, reduce distress, and promote wellbeing (Wu and Chung, 2023; Hilton, 2021). During times of solitude and crisis, creative engagement may offer both personal comfort and broader psychosocial value, extending beyond individual mental health to social and community wellbeing (Braus and Morton, 2020; Fancourt and Finn, 2019). In this sense, creative expression may function as an accessible coping resource during periods of heightened uncertainty. At the same time, conceptual precision is necessary. In much of the literature, expressive arts therapy refers to structured therapeutic work facilitated by trained professionals. Art therapists are expected to maintain multicultural and diversity competence to deliver ethically appropriate treatment interventions that are responsive to cultural context (Stepney, 2022). Similarly, art therapy may be combined with other therapeutic approaches, such as cognitive-behavioral therapy or mindfulness, as part of a broader treatment plan (Monson, 2022). However, the present study is concerned with women's own accounts of engaging in drawing, painting, movement, clay work, writing, and related creative activities as self-initiated practices during the pandemic, rather than with therapist-led clinical intervention. For this reason, terms such as expressive arts practices, creative expression, and arts-based coping strategies more accurately reflect the object of inquiry, even while the study remains informed by literature on expressive arts therapy as a broader field. Although previous studies have highlighted the benefits of art-related approaches for reducing anxiety and depression, and have discussed the growing relevance of expressive modalities for mental health support, important gaps remain (Wu and Chung, 2023; Hilton, 2021; Malchiodi, 2020). The literature has not sufficiently distinguished between formal therapeutic intervention and spontaneous, self-directed creative coping in everyday life. Moreover, limited attention has been given to the long-term role of such practices in supporting resilience and coping among working women in Malaysia. While pandemic mental health challenges have been widely discussed, less is known about how working women themselves describe the specific stressors they faced and how creative practices were incorporated into their coping routines. This gap is important because the experiences of working women are shaped not only by general pandemic uncertainty but also by gendered expectations, domestic labor, and occupational strain. A clearer understanding of these experiences can therefore contribute to the literature on women's mental health, coping, and creative expression in crisis contexts.

Accordingly, this study investigates how working women in Malaysia aged 21 to 60, who remained employed during the COVID-19 pandemic and experienced heightened occupational and domestic stress, used expressive arts practices to manage and cope with these pressures. Specifically, the study explores the types of stress they experienced, how creative and expressive practices were used in their daily lives, and how these practices were perceived in relation to stress management.

This study aimed to:

1. Explore the stress experiences of working women during the pandemic.
2. Examine how they engaged in creative or expressive activities to cope with stress.
3. Understand the perceived emotional and psychological effects of these practices.

### Expressive Arts Therapy (EAT)

Expressive Arts Therapy (EAT) has emerged as an innovative and holistic approach to stress management, particularly beneficial for those who might find traditional methods less effective (Malchiodi, 2020). According to (Braus and Morton 2020), COVID-19 and mental health difficulties are said to be at war, causing unrelenting stress as a consequence of the restrictions and guidelines put in place to slow the spread of the virus. Art Therapy may be utilized during times of solitude to assist people and may be used throughout one's whole lifetime. The benefits of Art Therapy extend beyond individual mental health, impacting social and community wellbeing (Fancourt and Finn, 2019).

### Theoretical underpinnings

This study is guided by two complementary strands of literature: coping theory and emotional regulation, and research on creative or expressive practices and psychological wellbeing. Lazarus and Folkman's Transactional Model of Stress and Coping, which conceptualizes stress as a dynamic transaction between the person and the environment. According to this perspective, stress arises not simply from external events themselves, but from how individuals appraise situational demands and evaluate their available coping resources (Lazarus and Folkman, 1984). The COVID-19 pandemic introduced multiple and overlapping demands for working women, including emotional strain, work-life disruption, uncertainty, and domestic pressures. From a coping perspective, participants' experiences may be understood in terms of how they interpreted these pressures and how they responded to them through personally meaningful coping strategies. In this study, creative and expressive activities are approached not as clinically prescribed interventions, but as coping practices that participants used to regulate emotions, manage distress, and restore a sense of balance in everyday life. Coping theory accommodates both the subjective experience of stress and the active processes through which individuals attempt to manage it (Lazarus and Folkman, 1984). Emotional regulation forms an important part of this process, particularly in contexts where stress is prolonged and difficult to resolve directly. During the pandemic, many stressors were ongoing and uncontrollable, which made emotion-focused coping especially relevant. (Jung 2014) argued that images and symbols can serve as important vehicles through which inner psychological experiences are expressed and made meaningful, particularly when such experiences are difficult to articulate directly in words. Activities such as drawing, painting, movement, clay work, and writing may therefore be understood as strategies through which participants expressed feelings, reduced emotional intensity, and created moments of calm, focus, or relief.

The second strand of the framework draws on literature concerning creative and expressive practices and their relationship to psychological wellbeing. Prior work suggests that art-related activities can support emotional expression, reflection, and stress reduction, particularly when individuals find it difficult to verbalize their inner experiences (Fancourt and Finn, 2019; Wu and Chung, 2023). The non-verbal and sensory qualities of creative practices may offer an accessible way of processing anxiety, grief, tension, and uncertainty. Existing literature has noted that arts-based engagement may contribute to emotional wellbeing and broader psychosocial support, especially during times of crisis and isolation (Malchiodi, 2020; Braus and Morton, 2020; Hilton, 2021). The present study examines self-initiated practices that participants incorporated into their own routines during a period of stress. For this reason, the current framework treats drawing, painting, movement, sculpting, and writing primarily as forms of creative expression or arts-based coping strategies. These practices may support psychological wellbeing by enabling emotional expression, bodily release, reflection, distraction from stress, and the cultivation of positive emotions.

## Method

This study adopted a qualitative design, guided by an interpretivist orientation (Braun and Clarke, 2006). Qualitative method was considered appropriate because the study aimed to generate a rich, experience-near account of participants' stressors and coping processes rather than to develop a formal theory or test predefined hypotheses, consistent with established approaches to qualitative inquiry and research design (Creswell and Poth, 2018). The data were analyzed using Braun and Clarke's (2006) reflexive thematic analysis. In line with this approach, the analysis involved repeated reading of the transcripts for familiarization, the generation of initial codes, the construction and refinement of candidate themes, the review and definition of themes, and the production of the final report. The analysis was interpretive and iterative, with themes developed through sustained engagement with participants' accounts rather than through a structured coding paradigm such as open and axial coding.

All participants provided informed consent before the initiation of data collection. Ethical approval for the study was obtained from the institutional ethics committee at Pusat Kecemerlangan Kesihatan Mental Kebangsaan Kementerian Kesihatan Malaysia, ensuring adherence to the principles of voluntary participation, confidentiality, and the opportunity to withdraw at any moment without repercussions. Participants were allocated pseudonyms to safeguard their identities, and all digital recordings and transcripts were securely archived. Expressive Arts Therapy was examined as a participant-initiated coping strategy that emerged organically in response to pandemic-related stress. The activities were autonomously undertaken and tailored by individuals according to their interests, available time, and emotional requirements. The data analysis adhered to Braun and Clarke's (2006) six-step thematic analysis framework, renowned for its rigor and flexibility in qualitative research. The phases encompassed data familiarization, first code development, topic identification, theme review, theme definition and naming, and final report production. Methodological rigor was enhanced through concrete procedures aligned with Lincoln and Guba's (1985) trustworthiness criteria. Credibility was supported through prolonged engagement with the interview transcripts, repeated reading during coding and theme development, and close grounding of interpretations in participants' own accounts. Trustworthiness was addressed using Lincoln and Guba's (1985) criteria of credibility, transferability, dependability, and confirmability. Credibility was supported through audio-recorded, in-depth interviews that were transcribed verbatim, and through iterative engagement with the dataset during coding and theme development, ensuring that themes were grounded in participants' accounts rather than in prior assumptions. Transferability was strengthened by providing contextual and demographic detail about the sample and study setting (e.g., age range, occupational diversity, marital and parenting status), enabling readers to judge the applicability of the findings to comparable contexts (Lincoln and Guba, 1985).

Dependability was enhanced by maintaining a clear and transparent analytic process, including documentation of interview procedures, coding decisions, and the progression from initial codes to final themes, thereby creating an audit trail of methodological and analytical steps (Lincoln and Guba, 1985). Confirmability was reinforced by retaining raw data, coded transcripts, and analytic memos, and by practicing reflexivity (e.g., documenting the researchers' assumptions and decision points) to demonstrate that interpretations were derived from the data rather than researcher preference (Lincoln and Guba, 1985).

### Data collection

The semi-structured interview guide was organized around the study objectives and included questions on participants' experiences of stress during the COVID-19 pandemic, the forms of creative or expressive practices they engaged in, how these practices were incorporated into their daily routines, and how they perceived their role in coping with stress. All interviews were conducted by the researcher through online video conferencing platforms in accordance with pandemic-related safety considerations. Each interview lasted approximately 45 to 90 mins, depending on the depth of participants' responses and the complexity of their experiences. With participants' consent, all interviews were audio-recorded and later transcribed verbatim for analysis. The interview guide was piloted with a small number of individuals who met the study profile to assess clarity, relevance, and sequencing of the questions. Minor refinements were made to improve wording and flow before the main data collection commenced.

### Participants descriptions

A sample of 31 participants was considered adequate for the present qualitative descriptive study because it enabled the generation of information-rich data across varied occupational and personal contexts while remaining manageable for in-depth reflexive thematic analysis. In qualitative inquiry, sample adequacy is commonly assessed in relation to the richness and relevance of the material rather than numerical representativeness. Consistent with concept of information power, the focused aim of the study, the specificity of the inclusion criteria, and the depth of the interview data supported the appropriateness of the final sample (Malterud et al., 2016). Participants were included if they met the following criteria: (1) female; (2) aged 21–60 years; (3) residing in Malaysia during the COVID-19 pandemic; (4) actively employed during the pandemic; (5) had engaged in expressive or creative practices as part of stress management during that period; and (6) were willing to participate voluntarily in an online semi-structured interview. Participants were excluded if they did not meet these criteria, had not engaged in such practices during the pandemic, or were unable or unwilling to provide informed consent. Table 1 shows the demographic profile of the Respondents.

**Table 1:** Demographic profile of the respondents.

Respondent ID	Age group	Occupation	Marital status	Children (Y/N)	EAT form(s)
R1	30–39	Teacher	Married	Y	Drawing/Coloring/Painting
R2	40–49	Healthcare professional	Married	Y	Drawing/Coloring/Painting; Dance/Movement
R3	20–29	Marketing specialist	Single	N	Dance/Movement
R4	50–59	Business owner	Divorced	Y	Dance/Movement; Clay/Sculpting
R5	20–29	IT analyst	Single	N	Dance/Movement
R6	30–39	Nurse	Married	Y	Drawing/Coloring/Painting; Dance/Movement
R7	40–49	University professor	Widowed	N	Dance/Movement
R8	30–39	Graphic designer	Married	N	Drawing/Coloring//Sculpting
R9	20–29	HR manager	Married	Y	Drawing/Coloring/Painting
R10	30–39	Social worker	Single	N	Dance/Movement
R11	40–49	Engineer	Married	Y	Drawing/Coloring/Painting
R12	20–29	Accountant	Single	N	Drawing/Coloring/Painting
R13	50–59	Retail manager	Divorced	Y	Dance/Movement
R14	20–29	Administrative assistant	Single	N	Drawing/Coloring/Painting; Dance/Movement; Clay/Sculpting
R15	30–39	Lawyer	Married	Y	Dance/Movement; Clay/Sculpting; Writing
R16	40–49	Pharmacist	Married	Y	Drawing/Coloring/Painting; Dance
R17	30–39	High school counselor	Single	N	Dance/Movement
R18	20–29	Journalist	Single	N	Clay/Sculpting
R19	30–39	Architect	Married	Y	Drawing/Coloring/Painting
R20	30–39	Sales executive	Married	N	Clay/Sculpting
R21	40–49	NGO worker	Single	N	Drawing/Coloring/Painting
R22	30–39	Financial analyst	Single	N	Clay/Sculpting
R23	40–49	School principal	Married	Y	Clay/Sculpting; Writing
R24	20–29	Software developer	Single	N	Drawing/Coloring/Painting
R25	30–39	Artist	Married	Y	Dance/Movement
R26	30–39	Event planner	Divorced	Y	Dance/Movement
R27	40–49	Environmental scientist	Married	N	Dance/Movement; Clay/Sculpting
R28	20–29	Fashion designer	Single	N	Clay/Sculpting
R29	50–59	Chef	Married	Y	Clay/Sculpting
R30	20–29	Publicist	Single	N	Dance/Movement; Clay/Sculpting
R31	30–39	Civil servant	Married	Y	Drawing/Coloring/Painting; Clay/Sculpting

## Findings

### Research objective 1—Explore the stress experiences of working women during the pandemic

The exploration of working women's experiences in managing stress through Expressive Arts Therapy during the COVID-19 pandemic revealed a complex array of challenges and coping strategies. The thematic analysis of the collected data identified five key themes: Navigating Mental and Emotional Turbulence, Struggling with Work-Life Integration, Financial Insecurity and Job Uncertainty, Loss and Grief, and Seeking Control in a Sea of Uncertainty.

#### Navigating mental and emotional turbulence

Navigating Mental and Emotional Turbulence emerged as a key theme in the respondents' experiences during the pandemic, highlighting the universal struggle of working women to cope with a range of mental health challenges. The sudden and pervasive impact of COVID-19 intensified feelings of anxiety, depression, and isolation, significantly affecting their psychological wellbeing. Respondents shared how the fear of the unknown, concerns about health and job security, and the constant barrage of distressing news led to a persistent state of mental stress:

“*During the pandemic, I felt… everything was uncertain.” R11*.

The respondents highlighted the compounded sense of loss, creating an undercurrent of grief that permeated the pandemic experience, challenging individuals' mental health resilience and coping mechanisms. In the aftermath of the COVID-19 pandemic, the respondents indicate that grief experiences that are particularly intense, prolonged, and potentially pathological are becoming more common:

“*I was worried about getting sick, worried about..my … my job, and,.. every day the news seemed to bring more bad updates.” R5*.

“*After some time, it felt like I was carrying a continuous mental issue that I could not… off.” R2*.

To comprehend the potential reasons for this, it was necessary to first identify the unique characteristics of pathological grief. The pandemic's pervasive impact on all aspects of life meant that grief was not only about the death of individuals but also about the symbolic death of certainty, predictability, and the future as once imagined.

“*At the same time, the stream of alarming news made it very difficult to feel calm, so the stress became persistent.” R7*.

Various terms, such as “complicated grief,” “traumatic grief,” and “persistent complex bereavement disorder,” have been proposed in recent years to refer to pathological grief. An intense yearning for or preoccupation with the deceased was the hallmark of a protracted grief disorder. This was accompanied by additional symptoms from a list that includes emotional numbness, a sense that life lacks meaning, disbelief, avoidance of reminders, categories of emotional pain, problems reintegrating into the social world, and loneliness or disturbance of identity. It was also claimed that prolonged grief was characterized by impaired social or occupational function, inconsistency with culturally anticipated responses, and inexplicability in relation to other established categories of mental disorder:

“*Even after many months, I still felt the loss very strongly. It was not just sadness—it felt like the grief stayed with me every day.” R30*.

“*I lost my relative during the pandemic, the shock was very hard to process. It felt sudden and overwhelming” R11*.

“*Sometimes it felt like life had changed completely, and I struggled to move forward. The feeling of loss stayed much longer than I expected.” R23*.

“*I thought I would recover with time, but the grief kept coming back, especially when I was alone or reminded of the situation.” R29*.

This multifaceted loss demanded new forms of resilience and adaptation, pushing individuals to seek alternative means of support and connection in the face of overwhelming sorrow. These terms are characterized by substantially overlapping diagnostic criteria.

#### Struggling with work-life Integration

The theme of Struggling with Work-Life Integration highlights a pervasive challenge faced by working women during the pandemic: the blurring of professional and personal spheres into an ongoing, indistinct cycle. Women found themselves caught in a relentless pursuit of efficiency, juggling professional responsibilities alongside household chores, childcare, and, for some, homeschooling. The absence of a structured work environment and the constant presence within the home space made it difficult to “switch off” from work, leading to extended hours and increased mental and emotional fatigue. Uncertainty has emerged as a theme in many people's lives during the pandemic:

“*Working from home made it very hard to separate my job and family responsibilities.” R14*.

“*It felt like there was no clear… [boundary] between work and home.” R8*.

“*I was juggling my job and household tasks continuously. I often felt mentally and emotionally drained.” R22*.

“*There was always uncertainty about what would happen next. That uncertainty added to my stress.” R28*.

Many respondents found themselves in positions with an uncertain future, particularly when they believe they are making the “smart” move and striving for stability and security. This meant creating strict schedules to delineate work from personal time for the respondents, despite the blurring boundaries of home and office spaces. Although many were aware of the majority of the uncertainty they encountered, it was still a source of stress.

“*I…I was doing everything carefully to stay stable, I still worried about what might happen next, especially with my work situation.” R25*.

“*but it was still difficult because everything was happening in the same space.” R17*.

For some, uncertainty has decreased as they have acquired the ability to remain composed in the face of the unknown and prevent the tension as they sought control through adopting new hobbies or routines that offered a sense of accomplishment and normalcy, even in small measures.

“*At first, the uncertainty was very stressful, but gradually I found ways to manage it by creating new routines that helped me stay organized and positive.” R3*.

“*Doing small activities every day helped me feel stronger emotionally. It reminded me that I could still manage my life despite the situation.” R9*.

The efforts to regain control, therefore, were not just about managing time or tasks but about reclaiming agency in one's life. This theme was a poignant reminder of the resilience and resourcefulness of individuals as they adapted to new realities, finding innovative ways to cope with the stress and uncertainty that defined the pandemic era.

#### Financial insecurity and job uncertainty

As businesses closed or reduced operations due to global lockdowns, many women faced job cuts, furloughs, or significant income reductions. This financial stress was further compounded by the unique pressures faced by women in the workforce. Many respondents detailed the additional burden of managing household expenses, childcare costs, and other domestic responsibilities without the usual support networks or resources.

“*I tried to …, but there was always a feeling that the future was uncertain, and that made me anxious.” R6*.

“*I was concerned about my health, my family's safety, and…. my job.” R1*.

As working women voiced their fears and uncertainties, their narratives called for a collective re-evaluation of the structures that underpin economic security and employment. Table 2 outlines the associated Themes and Codes to research objective 1.

**Table 2:** The associated themes and codes to research objective 1.

Research objective 1	Theme	Codes
To explore the type of stress experienced by working women during the COVID-19 pandemic	Navigating Mental and Emotional Turbulence	anxiety, depression, isolation, fear, emotional distress, pathological grief, symbolic loss, emotional numbness
	Struggling with Work-Life Integration	blurred boundaries, extended hours, multitasking, fatigue, routine creation, coping strategies, self-agency
	Financial Insecurity and Job Uncertainty	income loss, job insecurity, childcare burden, lack of support

### Research objective 2—To examine how they engaged in creative or expressive activities to cope with stress during the COVID-19 pandemic

The results show that emotional wellbeing and daily functioning of individuals were significantly impacted by anxiety disorders. This study confirmed the therapeutic potential of Expressive Arts Therapy in the reduction of stress and the improvement of coping skills in individuals with anxiety disorders. Themes emerged around the crucial aspects of Emotional Expression and Self-Exploration, highlighting Expressive Arts Therapy's role in providing a non-verbal medium for processing complex emotions and fostering self-discovery:

“*During that time, my anxiety affected how I felt every day. It was difficult to stay focused and manage my normal routine.” R25*.

“*I noticed that my stress and worry started to affect my mood and my ability to do daily tasks.” R2*.

“*There were days when my anxiety made it hard to concentrate on work or take care of things at home.” R16*.

Physical and Mental Relief underscored the immediate benefits of this method in alleviating bodily tension and clearing mental clutter, promoting a sense of calm and focus. The function of expressive arts was to offer a secure and non-verbal channel for stress, utilizing empirical evidence and theoretical foundations. The thematic analysis indicates that expressive arts were used by working women as a self-initiated coping process that operated through four closely connected pathways: Emotional Expression and Self-Exploration, Physical and Mental Relief, Increased Positive Emotions, and Strength and Psychological Stabilization. Participants described expressive arts as providing a non-verbal channel to externalize difficult feelings that were hard to articulate during the pandemic, enabling emotional clarification and deeper self-understanding through the creative process.

“*When I was drawing or writing, I could express feelings that I did not know how to say in words.” R13*.

“*The creative activities helped me understand my emotions better and reflect on what I was going through.” R30*.

“*Art became a way for me to release my feelings and make sense of my stress during the pandemic.” R2*.

At the same time, the sensory and embodied nature of activities such as drawing, painting, movement, and clay work supported immediate stress reduction by easing bodily tension, clearing mental “clutter,” and fostering present-moment focus.

“*When I was working with clay, my body started to relax, and I felt the tension slowly leaving my shoulders.”*

“*Doing these activities helped me clear my mind and focus on the moment.”*

“*The drawing or shaping clay, made me feel calmer and more settled emotionally.” R4*.

“*Using my hands in creative activities helped me release stress and feel more in control of my thoughts.” R19*.

These experiences contributed to longer-term outcomes characterized by enhanced resilience, emotional steadiness, and a renewed sense of personal agency, positioning expressive arts not only as short-term relief but also as a stabilizing resource for coping with sustained uncertainty and cumulative stress during COVID-19.

#### Emotional expression and self-exploration

The theme of Emotional Expression and Self-Exploration emerged in the respondents' narratives, highlighting its crucial role in managing stress and navigating emotional upheaval. Understanding self-expression was crucial for fostering emotional healing caused by Expressive Arts Therapy. It serves as a bridge connecting an individual's internal state with their external experience. As respondents immersed themselves in art-making, they often accessed feelings that were difficult to name, such as fear, grief, isolation, or emotional exhaustion and gave those experiences shape through concrete creative actions. For example, some women used drawing or coloring (e.g., repetitive patterns or simple sketches) to quiet anxious thoughts and regain a sense of control; others used painting to “release” intense emotions through color, strokes, or imagery when words felt insufficient; dance and movement were described as ways to discharge tension and express emotions physically; and clay work or sculpting provided a tactile outlet where squeezing, shaping, or molding became a grounding form of emotional processing. In some cases, writing functioned as an expressive outlet akin to emotional journaling, allowing participants to translate distress into narrative meaning and personal insight:

“*Sometimes I felt emotions that I could not clearly explain, but when I started creating something, those feelings became easier to understand.” R15*.

“*Art really helped me recognize emotions like fear and sadness that I did not know how to describe before.” R1*.

“*Through creative activities, can help to manage stress.” R20*.

As respondents immersed themselves in various art forms, they tapped into unexplored aspects of their psyche, uncovering emotions and thoughts that were previously unacknowledged or misunderstood. Moreover, Self-expression encompasses various modes, including verbal and non-verbal forms. Some participants encountered a spectrum of feelings, ranging from fear and anxiety to grief and isolation, that were challenging to express through words alone.:

“*Sometimes I knew I was anxious or upset, but I did not have the right words to describe how I felt.” R14*.

“*There were moments when my emotions felt overwhelming, and talking about them was not easy.” R23*.

The tactile experience of artmaking, the feel of paint on canvas, the rhythm of a dance, and the sensation of clay between fingers engaged the body in a way that verbal expression could not, grounding individuals in the present moment and fostering a sense of mindfulness:

“*Moving my body to music helped me express my emotions and feel more relaxed.” R6*.

“*When I danced, the rhythm of my movements helped release tension and made my body feel lighter.” R1*.

This embodied approach to emotional exploration helped to bridge the gap between physical sensations and emotional states, encouraging a more integrated sense of self.

#### Physical and mental relief

The respondents stressed that the act of engaging in artistic activities, whether it be through the tactile experience of painting, the rhythmic movements of dance, or the focused attention required in sculpting, offered significant physical and mental benefits. Art Therapy was a practice that was remarkably adaptable and could be employed in a variety of ways to promote mental health. A mental health care provider may employ a variety of techniques to assist their client, contingent upon the individual's requirements and their prior experiences with therapy. Participants reported feeling a notable reduction in physical stress symptoms, such as tightness and discomfort, as they immersed themselves in the sensory experiences of their chosen art forms. The respondents stated that the objective of Art Therapy was to assist in the development of a more profound sense of self-awareness through creative expression, which in turn enabled them to acquire supplementary coping mechanisms that can alleviate tension or alleviate symptoms of anxiety or depression. Coloring, drawing, music, photography, sculpture, and writing are among the most frequently employed Art Therapy techniques:

“*When I was painting, the feeling of it…helped me focus and forget my worries for a while.” R10*.

“*Touching and shaping the clay with my hands made me feel calmer and more connected to what I was doing.” R15*.

“*The physical act of creating something helped me relax because my attention was on the movement and the material.” R21*.

In addition to the creative process of creating art, individuals may also acquire additional insights through reflection that can assist them in progressing in their lives. The numerous advantages of Art Therapy are substantiated by the inherent connection between art and the brain. The integration of body and mind in the creative process facilitated a comprehensive healing experience, demonstrating the interconnectedness of physical and mental wellbeing. The practice of Expressive Arts Therapy became a mindful activity, encouraging participants to be present in the moment, fully engaged in the creative process, and temporarily detached from the ongoing stresses and anxieties of pandemic life. This shift in perspective underscored the interconnectedness of mental, emotional, and physical health, highlighting the need for a balanced approach to wellbeing that incorporates creative expression. In challenging times, Expressive Arts Therapy offered a pathway to resilience, demonstrating that through self-care and mindfulness, individuals could navigate the complexities of their emotional landscapes with grace, finding solace and strength in the act of creation.

#### Increased positive emotions

The theme of Increased Positive Emotions through the engagement with Expressive Arts Therapy captures a significant shift experienced by participants during the COVID-19 pandemic. Amidst a backdrop of uncertainty and stress, activities such as dancing, painting, and other creative expressions served as powerful conduits for emotional transformation. The act of bringing one's inner world to life through art provided a tangible sense of accomplishment and satisfaction, countering feelings of despair and helplessness that the pandemic often engendered. The process of engaging in Expressive Arts Therapy fostered an environment where positivity could flourish. This mindfulness, in turn, opened the door to experiencing more positive emotions, as individuals found themselves absorbed in the creative process, experiencing flow and the intrinsic pleasure of artistic expression. According to the participants, the Therapy was not merely about taking a break; it was about rediscovering moments of happiness and delight amid adversity. Engagement in Expressive Arts Therapy elicited a range of positive emotions that supported participants' overall sense of wellbeing and helped counterbalance the emotional gloom associated with pandemic-related stress. Beyond joy and happiness, participants commonly described affective shifts characterized by calmness and relief, feelings of comfort or being soothed, renewed hopefulness, gratitude, and moments of interest and curiosity during the creative process. Respondents also reported satisfaction linked to completing an artwork or sustaining a routine, alongside increased confidence, contentment, and a sense of renewed energy or enthusiasm:

“*After spending time on creative activities, I felt calmer and more relaxed, like the stress had eased for a while.” R23*.

“*Working on my artwork gave me a sense of a bit comfort during difficult days.” R12*.

“*yes. It helped me feel relieved and more emotionally settled.” R14*.

“*Doing something creative gave me hope that things would get better, even during uncertain times.” R24*.

“*I could still find moments of peace despite the challenges.” R17*.

In this study, such positive emotions are understood as immediate, experience-based affective states, whereas resilience is more appropriately conceptualized as a longer-term psychological capacity or outcome that may be strengthened through repeated engagement in these practices.

The positive emotions elicited through artistic expression contributed to an overall sense of wellbeing, offsetting the gloom and helping individuals maintain a more balanced emotional state. The theme also underscores the broader implications of Expressive Arts Therapy as a source of distraction and joy for mental health. In navigating the emotional challenges of the pandemic, the ability to find moments of happiness and engagement through art underscored the resilience of the human spirit. The theme also captures the transformative power of Expressive Arts Therapy in navigating the emotional complexities wrought by the pandemic. This alchemical process, where art becomes a medium for transmuting difficult emotions into meaningful and beautiful creations, provided individuals with a potent mechanism for coping with and understanding their internal landscapes.

#### Strength and psychological stabilization

As individuals explored their emotions and experiences through the creative process, they often discovered inner resources of strength and resilience they were previously unaware of. The sense of accomplishment and control gained from creating something tangible, be it a piece of art, a dance routine, or a series of written words, reinforced their confidence in their ability to influence their environment and outcomes, an essential component of psychological resilience.

“*I think, creative activities … helped me feel more emotionally stable, not just during the activity but throughout my daily life.” R1*.

“*These activities helped me stay calmer and more balanced, even when new challenges appeared.” R24*.

“*I was,..more patient after creative practices part of my routine.” R8*.

The stabilization of psychological states through Therapy extends beyond individual moments of art-making to influence participants' overall approach to life and wellbeing. Table 3 outlines the associated Themes and Codes to research objective 2.

**Table 3:** The associated themes and codes to research objective 2.

Research objective 2	Theme	Codes
To understand how expressive Art Therapy is used in managing stress among working women during the COVID-19 pandemic	Emotional Expression and Self-Exploration	Non-verbal expression, emotional clarity, inner exploration
	Physical and Mental Relief	Bodily relief, mental clarity, relaxation, flow, the integration of body and mind, a comprehensive healing experience, and interconnectedness of physical and mental wellbeing.
	Increased Positive Emotions	Joy, satisfaction, positive outlook, artistic flow, self-compassion, mindfulness, present-moment focus
	Strength and Psychological Stabilization	Resilience, inner strength, emotional stability, stabilization of psychological states

### Research objective 3—To understand the perceived emotional and psychological effects of these practices in managing stress during the COVID-19 pandemic

The responses highlight a range of creative activities, including drawing, coloring, painting, dancing, working with clay, and writing. These practices provided individualized ways for participants to address the emotional and psychological challenges brought on by the pandemic. The data reveal that participants incorporated expressive art activities into their routines with differing regularity, mostly influenced by their work obligations, resource availability, and previous involvement in artistic endeavors:

“*I chose the ones that suited my mood and situation.” R2*.

“*I tried several forms of creative expression.” R10*.

“*It helped me handle the emotional pressure of the pandemic.” R24*.

“*Some weeks I practiced creative activities more often, especially when work stress was high.” R9*.

“*I tried to include creative time in my routine, but the frequency depended on my work schedule and responsibilities.” R28*.

“*When things were calmer, I spent more time on creative activities.” R20*.

Of the 31 working women interviewed, twenty-five engaged in these activities weekly, and some reported practicing them multiple times per week, particularly during periods of heightened stress or lockdown. Each session lasted between about 30 min and two hours, demonstrating the participants' adaptability in aligning the routines with their personal schedules and emotional requirements.

#### Drawing and sculpting

Drawing and coloring emerged as significant therapeutic practices for many working women during the COVID-19 pandemic, offering not only a means of creative expression but also a crucial coping mechanism amidst the unprecedented stress and isolation.

“*These activities helped me manage my emotions when I felt overwhelmed and isolated.” R6*.

“*Spending time coloring gave me a break from stress and helped me feel more relaxed.” R29*.

The act of drawing and coloring offered moments of tranquility and solitude that became essential for mental wellbeing amidst the chaos of the pandemic. In the moments of artistic engagement, the participants found an opportunity for self-care and emotional healing. The repetitive act of coloring and shading self-drawn sketches and patterns and the sustained concentration involved in sketching functioned as grounding practices, helping to ease anxiety and foster a sense of inner calm:

“*When I was drawing, I could express my feelings without needing to explain them to anyone.” R10*.

“*Coloring helped me stay calm and focused, especially during long periods at home.” R13*.

The process of sculpting and working with clay acted as a dynamic medium for expressing creativity and exploring emotions in a non-verbal manner. This act of creation provided an outlet for emotional release, allowing individuals to externalize complex feelings that might be difficult to articulate through words alone. The engagement with tactile art forms like clay and sculpting also offered a break from the digital saturation and virtual interactions that became the norm during the pandemic. This return to a more tangible form of interaction and expression helped alleviate the sense of isolation and digital fatigue, contributing to overall wellbeing.

#### Dance and movement

The physical embodiment of emotions through dance provided a cathartic release, enabling individuals to process and, in some cases, convert negative feelings into empowerment, resilience, and even moments of joy. Furthermore, dance and movement practices provided an essential escape from the monotony and restrictions imposed by lockdown measures, reaffirming the body's role as a crucial instrument of self-expression and healing. In a time when physical spaces were limited and social interactions were minimized, dancing offered a unique avenue for reclaiming agency over one's body and space:

“*Moving my body to music made me feel happier and lifted my mood during difficult days.” R2*.

“*Dance gave me small moments of happiness”*

“*During the lockdown, ther was no physical activities, but dancing helped have some.” R23*.

“*Staying at home for long periods was tiring, and dancing became my way of escaping the boredom.” R10*.

As participants engaged in dance, they not only navigated their emotional landscapes but also experienced the physical benefits associated with regular exercise, such as improved mood, increased energy levels, and reduced stress. This approach to wellbeing, recognizing the body as an ally in the journey toward emotional and psychological health, underscores the adaptability and resilience of the human spirit (Chen et al., 2024).

#### Creative projects

Painting and other forms of artistic creation emerged as pivotal practices for individuals seeking to navigate the emotional tumult. The act of translating emotions into visual forms provided a tangible way to engage with and understand these feelings, facilitating a form of emotional journaling that was both cathartic and enlightening. Immersing oneself in the creative process allowed for a temporary detachment from the realities of lockdowns, social distancing, and the omnipresent sense of uncertainty that defined much of this period. This immersion in art offered not just a distraction but a profound engagement with the creative self, enabling individuals to lose themselves in the flow of creation. Creation became a powerful tool for emotional resilience, offering not only a way to manage stress and anxiety but also a pathway toward personal growth and healing.

The majority of participants indicated minimal or informal prior engagement with expressive art activities before the Pandemic began. For instance, although some individuals had previously engaged in informal drawing or painting, these activities had not been incorporated into their stress management strategies.

“*I used to draw sometimes when I had free time, but I never thought of it as a way to handle stress.” R15*.

“*Art was something I enjoyed casually, but I did not rely on it for emotional support before the pandemic.” R13*.

The pandemic environment intensified the demand for emotional release, leading many of these women to re-engage with or freshly embrace creative expressions as therapeutic outlets. A minority of participants possessed prior experience with artistic techniques via school or community workshops; nonetheless, formal art training was infrequent within the sample. This indicates that expressive Art Therapy does not necessitate professional creative ability or formal guidance to be efficacious. Its significance resides in its accessibility and adaptability, allowing women to intuitively connect with their emotions through diverse sensory and symbolic expressions. The therapeutic advantage seems to arise from consistent participation and the liberty to express oneself without the limitations of aesthetic evaluation, rather than from proficiency in any particular creative form (Khalil and Demarin, 2024). Table 4 outlines the associated Themes and Codes to research objective 3.

**Table 4:** The associated themes and codes to research objective 3.

Research objective 2	Theme	Codes
To evaluate how expressive Art Therapy is practiced by working women in managing stress during the COVID-19 pandemic	Drawing and Sculpting	Calming repetition, tactile engagement, grounding, reflection, processing, vocal expression, drawing, painting, making sculptures
Dance and Movement	Emotional release, embodiment, energy uplift
Creative Projects and Family Activities	Emotional journaling, creative flow, self-growth

## Discussion

As articulated by (Ciasca et al. 2018), the sensory engagement offered by various art forms not only serves as an emotional release but also plays a crucial role in alleviating the physical tension that accumulates in response to prolonged periods of stress and anxiety. The transition to remote work during the COVID-19 pandemic has significantly blurred the lines between professional and personal lives, particularly for working women, who have faced unique challenges in integrating these two spheres. This phenomenon, explored in the literature by (Bosgraaf et al. 2020), underscores the struggle to maintain productivity in a home environment while simultaneously managing domestic responsibilities, childcare, and, in some cases, home-schooling. The dissolution of physical boundaries traditionally separating work from home life has led to extended work hours and increased mental and emotional fatigue. As articulated in the work by (Wang et al. 2020), the pandemic has dramatically altered the fabric of everyday life, leading to a collective mourning process for a world that no longer exists in its previous form. The enforced isolation and disruption of traditional mourning rituals have further compounded this grief, leaving many to navigate their sorrow alone, without the customary supports that help people process and cope with loss.

The reflective nature of artmaking provides a safe space for individuals to confront their experiences and emotions, thereby fostering a sense of empowerment and emotional enrichment (Yang et al., 2021). According to (Yaribeygi et al. 2017), the act of creation inherent in Expressive Arts Therapy practices facilitates a meaningful interaction with one's emotional state, thereby enabling the transformation of negative emotional experiences into one's characterized by joy, peace, and overall wellbeing.

Although Arts Therapy is often discussed in relation to increased positive affect and psychological uplift (Yang et al., 2021), it is equally important to recognize that arts-based processes provide a safe and structured avenue for engaging with difficult emotions. In this study, participants' creative engagement can be conceptualized as a form of emotional processing in which distressing experiences (e.g., anxiety, grief, uncertainty, and emotional overload) are externalized into images, movement, or words, making them more approachable and tolerable. Through this non-verbal and embodied medium, challenging emotions can be explored with psychological distance, held safely within the creative act, and gradually accepted as part of the individual's experience, rather than suppressed or bypassed (Ben-Tzur and Feniger-Schaal, 2024; Czamanski-Cohen and Weihs, 2023). This view aligns with trauma- and stress-informed perspectives on expressive arts, which emphasize the role of creative work in regulating affect, integrating difficult internal states, and supporting adaptive coping and meaning-making, particularly under crisis conditions such as the COVID-19 pandemic.

This transformative process is not only therapeutic but fundamentally alters the emotional landscape of individuals, offering them a tangible sense of achievement and counteracting the pervasive feelings of despair brought about by the pandemic's challenges. Moreover, the role of Expressive Arts Therapy in fostering a conducive environment for the flourishing of positive emotions underscores its efficacy in enhancing mental health resilience among participants. The emphasis on present-moment engagement, as discussed by (Phillips and Becker 2019), whether through dance or visual arts, cultivates a state of mindfulness that encourages an appreciation of the current experience, minimizing the tendency to dwell on past adversities or future uncertainties. As highlighted by (Liddle et al. 2012), the act of creating art offers a tangible sense of accomplishment and a method for individuals to affirm their control over their environment and emotional state, crucial for maintaining a balanced psychological state amidst the turbulence of a global crisis. This aligns with the findings of (Safrai 2013), which underscore the importance of regular engagement with expressive arts as a source of psychological comfort and stability, illustrating the profound impact of Art Therapy in facilitating a journey toward enduring resilience and an optimistic outlook on life, despite the ongoing challenges posed by the pandemic.

Earlier studies reported that people turned to artistic and creative activities during lockdown periods to support their emotional wellbeing and maintain routine during periods of social isolation. For example, research conducted during the COVID-19 pandemic found that engagement in artistic creative activities was associated with emotional regulation, stress management, and resilience among participants facing prolonged uncertainty (Fancourt and Finn, 2019). Similarly, qualitative work examining coping strategies during the pandemic demonstrated that individuals relied on meaningful daily practices, including creative expression, to manage psychological distress and maintain a sense of stability (Bu et al., 2020). In this sense, the current findings align with a growing body of literature suggesting that creative engagement may be perceived as a personally valuable coping resource during crisis, while extending this discussion to the specific experiences of working women navigating pandemic-related stressors. Research conducted in Malaysia similarly reported that women working from home experienced increased role overload and emotional fatigue as they navigated simultaneous work and family demands during lockdown conditions (Adisa et al., 2021).

### Research implications

The findings highlight EAT's versatility and effectiveness, providing compelling evidence for its integration across various domains to strengthen psychological resilience and emotional wellbeing. This approach ensures that future therapists can personally experience the benefits of expressive arts, which is crucial for them to authentically integrate these practices into their professional repertoire. Facilitating workshops and practical sessions within these courses can foster a deeper understanding of the non-verbal communication methods and emotional processing techniques unique to Art Therapy. The findings from the study make important contributions to the theoretical frameworks surrounding stress management and emotional resilience. One of the key theoretical implications is the validation and extension of existing theories regarding the therapeutic power of creative expression.

### Limitations and future research recommendations

This study should be interpreted in light of several limitations. First, the sample was relatively small and consisted of 31 working women, which is appropriate for an in-depth qualitative inquiry but limits the breadth of perspectives that can be captured. As such, the findings are not intended to be statistically generalisable, but rather to provide a context-specific and experience-based understanding of how participants described stress and the use of expressive arts practices during the COVID-19 pandemic. The use of purposive recruitment through social media, online community forums, and professional networks may have introduced selection bias. Women who chose to participate may have been more open to discussing mental health, more interested in creative practices, or more likely to view such practices positively than those who did not volunteer. The sample may therefore underrepresent working women who did not engage in expressive activities or who experienced stress in different ways. Moreover, the study relied on self-reported accounts collected through interviews. While this approach was appropriate for exploring lived experiences and personal meanings, participants' responses may have been influenced by memory, personal interpretation, or a desire to present their coping strategies in a favorable light. The findings therefore reflect participants' perceptions and retrospective accounts rather than independently verified behavioral or psychological outcomes. Finally, because this study employed an exploratory qualitative design, it cannot establish causal relationships or confirm therapeutic effectiveness. The findings do not demonstrate that expressive arts practices directly reduced stress or improved mental health outcomes. Rather, they indicate how participants perceived and described these practices as meaningful coping resources during a period of uncertainty and disruption. Future research may build on these findings by using mixed-methods or longitudinal designs to examine these experiences across broader populations and over time.

## Conclusion

This study explored how working women in Malaysia experienced stress during the COVID-19 pandemic and how they used creative and expressive practices as part of their coping efforts. The findings highlight the complex emotional, occupational, and domestic pressures participants faced during this period, including anxiety, uncertainty, work–life strain, and emotional exhaustion. Participants described activities such as drawing, painting, movement, clay work, and writing as meaningful outlets for emotional expression, reflection, relief, and routine-building. Rather than demonstrating therapeutic effectiveness, the findings suggest that creative and expressive practices may represent meaningful coping resources for some women during times of crisis and disruption. The study also underscores the importance of recognizing the gendered dimensions of stress and the need for supportive mental health promotion strategies that are accessible, flexible, and sensitive to women's lived realities.

## Data Availability

The raw data supporting the conclusions of this article will be made available by the authors, without undue reservation.
